# Perceptions of Alerts Issued by Social Media Platforms in Response to Self-injury Posts Among Latinx Adolescents: Qualitative Analysis

**DOI:** 10.2196/28931

**Published:** 2021-08-10

**Authors:** Linnea I Laestadius, Katherine A Craig, Celeste Campos-Castillo

**Affiliations:** 1 Zilber School of Public Health University of Wisconsin-Milwaukee Milwaukee, WI United States; 2 Department of Sociology University of Colorado Boulder Boulder, CO United States; 3 Department of Sociology University of Wisconsin-Milwaukee Milwaukee, WI United States

**Keywords:** adolescents, social media, mental health, NSSI, race and ethnicity, mobile phone

## Abstract

**Background:**

There is growing interest in using social media data to detect and address nonsuicidal self-injury (NSSI) among adolescents. Adolescents often do not seek clinical help for NSSI and may adopt strategies to obscure detection; therefore, social media platforms may be able to facilitate early detection and treatment by using machine learning models to screen posts for harmful content and subsequently alert adults. However, such efforts have raised privacy and ethical concerns among health researchers. Little is currently known about how adolescents perceive these efforts.

**Objective:**

The aim of this study is to examine perceptions of automated alerts for NSSI posts on social media among Latinx adolescents, who are at risk for NSSI yet are underrepresented in both NSSI and health informatics research. In addition, we considered their perspectives on preferred recipients of automated alerts.

**Methods:**

We conducted semistructured, qualitative interviews with 42 Latinx adolescents between the ages of 13 and 17 years who were recruited from a nonprofit organization serving the Latinx community in Milwaukee, Wisconsin. The Latinx population in Milwaukee is largely of Mexican descent. All interviews were conducted between June and July 2019. Transcripts were analyzed using framework analysis to discern their perceptions of automated alerts sent by social media platforms and potential alert recipients.

**Results:**

Participants felt that automated alerts would make adolescents safer and expedite aid before the situation escalated. However, some worried that hyperbolic statements would generate false alerts and instigate conflicts. Interviews revealed strong opinions about ideal alert recipients. Parents were most commonly endorsed, but support was conditional on perceptions that the parent would respond appropriately. Emergency services were judged as safer but inappropriate for situations considered lower risk. Alerts sent to school staff generated the strongest privacy concerns. Altogether, the preferred alert recipients varied by individual adolescents and perceived risks in the situation. None raised ethical concerns about the collection, analysis, or storage of personal information regarding their mental health status.

**Conclusions:**

Overall, Latinx adolescents expressed broad support for automated alerts for NSSI on social media, which indicates opportunities to address NSSI. However, these efforts should be co-constructed with adolescents to ensure that preferences and needs are met, as well as embedded within broader approaches for addressing structural and cultural barriers to care.

## Introduction

### Background

The rates of nonsuicidal self-injury (NSSI) and suicidal ideation have been rising among adolescents in the United States, with Latinx adolescents experiencing high rates of both [1-3]. NSSI, also known as self-harm or self-injury, is defined as “deliberate, self-inflicted damage of body tissue without suicidal intent and for purposes not socially or culturally sanctioned” [4]. Although distinct in intent, NSSI is a risk factor for suicidality [5,6]. There is evidence that social media users share web-based NSSI content [7,8], creating interest in approaches using social media data to detect and address NSSI and prevent suicidality [9-13].

One approach, termed digital phenotyping, suggests that analysis (usually via machine learning) of data generated from interactions with digital technologies and devices, including social media, can be used to “identify and diagnose health conditions” [14-16]. Although initially envisioned to include clinician-generated inputs, such as medical records, the approach is now regularly applied to social media data without further inputs [10,16-19]. As adolescents often do not seek clinical help for NSSI [20] and may adopt strategies to obscure detection [7,21-23], the creation of alerts informed by digital phenotyping of social media data could provide new opportunities for early detection and treatment.

Although a potentially promising intervention, digital phenotyping alerts based on machine learning algorithms to detect mental health concerns raise questions about consent, efficacy, and privacy risks [14,24]. For example, Facebook uses digital phenotyping to detect suicidal ideation in posts made by US users and then alerts local emergency responders when the staff deems it is appropriate [13]. There is no way to opt into or out of this feature, even for minors [25]. Some have also raised questions about the accuracy of the underlying machine learning model analyzing posts to infer mental health status, particularly in cases where predictions are primarily built using data from adults [26]. Both false negatives and false positives weaken the efficacy of the intervention and may pose risks to adolescents. Although adults have shown little support for these efforts [27], adolescents’ perceptions of both alerts based on their social media posts and the commercial and institutional uses of personal data remain largely unknown [28]. Given the initial evidence that youth are less likely than adults to view their health information as sensitive and the collection of their personal data by companies as a privacy violation [29], they may be apathetic toward these interventions. However, the only prior study of the perceptions of young adults of digital phenotyping (which focused on assessing mental health via cellphone sensor data) demonstrated that they had concerns about privacy and autonomy and rejected the monitoring of text message content [30].

As interventions informed by social media data are already deployed and will likely spread to an increasing number of settings, it is an ethical and design imperative to understand how adolescents perceive these efforts and how mental health alerts based on digital phenotyping can best be tailored to meet their preferences and needs [12]. Prior research has stressed the importance of user-centered design and the cocreation of digital mental health tools with youth [25,30]. Moving ahead without considering youth perspectives may result in both rights violations and backlash against existing and future interventions [25]. For example, the only way adolescents may opt out of Facebook’s suicide intervention efforts is to discontinue using the Facebook and Instagram platforms, which may have negative implications for adolescents and would limit the completeness of data used to inform subsequent interventions. Accordingly, assessing youth perspectives on how interventions are designed may help improve their efficacy and accuracy.

### Study Aims

We address this gap in the literature by qualitatively examining how Latinx adolescents perceive social media platforms automatically detecting posts about NSSI and alerting an authority figure in response (*automated alerts*). We focus on Latinx adolescents because although they engage in NSSI, they are underrepresented in research on NSSI and are more likely to be undiagnosed than White adolescents [31]. Latinx adolescents also experience higher rates of depression, which is a risk factor for NSSI [32,33], than non-Hispanic White adolescents [2,32-34]. The actual rates of depression may be higher because clinicians may miss cultural differences in the expression of symptoms [35]. Thus, population-level models may introduce cultural bias into digital phenotyping efforts to infer NSSI [36]. At the same time, Latinx adolescents may stand to benefit from such tools because of socioeconomic and cultural barriers to receiving mental health screening [37]. Moreover, the Latinx community is underrepresented in studies of privacy on social media [38-40] despite factors such as documentation status and larger household sizes, potentially creating unique privacy needs and ethical concerns [41]. In the context of mental health, potential stigmatization shapes who they are likely to approach for mental health support [42] and thus may also influence their preferences for who should receive an alert. Accordingly, it is critical to engage with Latinx adolescents to study how they perceive interventions that automatically alert a third party to obtain help when adolescents make concerning social media posts and their preferences for alert recipients.

## Methods

### Data Collection

Eligible participants were Latinx adolescents aged 13 to 17 years who used social media, defined as posting at least once per week. We collaborated with a community organization serving the Latinx community in Milwaukee, Wisconsin, which hosts a summer youth program to recruit and screen participants based on the eligibility criteria. Adolescents gave assent, and guardians provided consent before data collection proceeded. Both assent and consent documents were available in English and Spanish. Although the family origin of students at the community organization was not available, Milwaukee’s Latinx population is largely of Mexican descent [43]. Participants who were enrolled in the study received a US $40 incentive. The institutional review board at the University of Wisconsin-Milwaukee approved the research protocol.

Between June and July 2019, 60- to 90-minute semistructured, in-person interviews were held with 43 Latinx adolescents in a quiet room at the community organization. Although a Spanish-speaking interviewer was available, all interviews were conducted in English at the request of the adolescents. At the start of the interview, participants provided demographic information and selected a study pseudonym. As part of a broader line of questioning about how adolescents handle mental health disclosures from peers on social media [44], participants were asked the following question: “How would you feel if a social media app sent an automatic alert to an adult when you or your friends made a post about self-harm?” The term self-harm was chosen over NSSI to reflect common use [7]. The participants were then asked who should receive such alerts. We probed about emergency services, which the Facebook suicide intervention alerts, and *trusted adults* (parents or guardians and school staff or teachers) frequently identified in prior literature [44,45]. Participants were also probed about why they endorsed or opposed these alert recipients. One interview concluded without asking about automated alerts because of time constraints, resulting in a sample size of 42 participants.

### Data Analysis

Following professional verbatim transcription, the interdisciplinary team, consisting of a senior qualitative researcher in public health, a senior sociologist who is Latinx, and 2 graduate students trained in qualitative methods, analyzed the transcripts using framework analysis [46] to identify perceptions of automated NSSI alerts and alert recipients. The 2 senior members performed open coding on the transcripts for the first 12 interviews to identify the initial codes and develop the codebook. The codebook captured perceptions for and against automated alerts. Transcripts were then split among team members and coded using MAXQDA 2018 (VERBI Software GmbH). Throughout the final coding process, an additional 11 transcripts were coded by 2 to 3 members and discussed code by code to allow new codes to arise and ensure a shared understanding of code definitions. Any discrepancies were resolved through consensus, with prior coding updated as needed using an iterative process [47]. Coded transcripts were then charted into a framework analysis matrix [46] using MAXQDA 2018 to summarize data by category from each transcript, with a focus on identifying favorable and unfavorable perceptions of automated alerts and alert recipients. In addition, memos were written to aid in the data interpretation and identification of representative quotes [48].

## Results

### Overview

Of the 42 participants, 24 (57%) self-identified as girls and 18 (43%) as boys. The ages ranged from 13 to 17 years, with a mean age of 15. Over 61% (26/42) of the participants indicated that both of their parents were immigrants in the United States. The participants most frequently used Snapchat (42/42, 100%), followed by Instagram (39/42, 93%). We present findings on the perceptions of automated alerts for NSSI, followed by the preferred recipient of such alerts. The majority of participants felt that automated alerts would make adolescents safer, but also expressed strong concerns about alert accuracy and the efficacy of alert recipients. The representative quotes below are presented using the pseudonyms selected by the participants.

### How do Latinx Adolescents Perceive Automated Alerts for NSSI?

Participants worried about whether digital phenotyping tools could accurately detect NSSI, specifically mentioning false positives due to (1) the frequent use of jokes, sarcasm, and hyperbole by adolescents, and (2) situations that were seen as posing only minor levels of risk that did not necessitate intervention. For example, Dahlia (age 14) supported alerts but worried about social media posts being misinterpreted, and alerts sent inappropriately:

I think I would be okay with that because in the end they’re just trying to help, but at the same time if like you were saying that but you didn’t really mean that and someone were to find out, that would be like embarrassing.

Ariona (age 14) similarly worried about common turns of speech being taken literally and parents overreacting to an alert:

...*if it was just joking around, your parents might take it way too seriously.* [Ariona]

*So what’s an example of someone joking around about self-harm?* [Interviewer]

*Like sometimes when we have a bad test that was really hard and we’ll be like, “I want to kill myself,” but it’s just joking. We’re not being for real about it.* [Ariona]

Fears also included getting in trouble with authorities if emergency services were summoned without an emergency being present.

Pablo (age 17) explained as follows:

Don’t you get like fined and stuff like that like if the ambulance comes?...What if the algorithm gets it wrong for some reason then you get some ambulances outside your house?

Broad privacy concerns were relatively rare and focused primarily on the desire for adolescents to tell adults themselves rather than an impersonal alert being sent on their behalf. Although none of the participants indicated that they would stop posting to regain control of their privacy, a small number anticipated that other adolescents may do so. More commonly, participants dismissed privacy concerns as minor relative to the severe risks posed by NSSI. Although the interview questions about automated alerts focused on self-harm, participants often linked NSSI with suicidality, both conceptually (as with the quote from Ariona above) and with regard to the importance of timely intervention to curtail escalation. Perceptions of urgency were the primary drivers of support for automated alerts. Ashley (age 15) explained as follows:

Like it’s good for [parents] to get that alert just so you don’t do anything more stupid than trying to self-harm yourself, and so that way your parents can know on time to like hurry and like tell you before you do anything else.

Similarly, David (age 17) felt alerts would “probably be a good thing because it’d probably alert the parent or guardian before the harmful thought got too serious.”

Participants also noted that adolescents often post about mental health struggles without anyone acting on it and expressed a strong sentiment that parents or guardians are commonly unaware of how their children are feeling. Bri (age 17) felt that alerts were as follows:

pretty smart, because so many people tend to stay quiet. They tend to keep it on social media. They usually don’t tell their parents, hey, this person that I know is feeling like this. And it’s just better to be safe than sorry...It’s better to do that than say that and then no one says anything, and then it just stays in that toxic state. Like it’s just there. You keep saying you’re depressed, and no one says anything. And something could happen.

Consequently, participants felt that adolescents struggling with NSSI may experience barriers to timely help. Automated alerts were seen as faster and more reliable for obtaining aid than waiting on friends to take action or parents or guardians to notice. Alerts were mentioned as a means of improving communication among adults and children. Once notified, participants generally anticipated that adults would offer adolescents some form of mental health support. Monse Marie (age 13) explained this as follows:

I think that would be a good thing for the social media to do because if you’re going through something then the parent will try to help you like oh, maybe therapy or something, you know, besides like because sometimes parents are blinded, like they don’t know what’s going on with the kid.

### Who Should Receive Automated Alerts for NSSI?

Despite strong support for automated alerts because they could yield adult help, participants were cognizant of adults differing in their efficacy and desire to help. An alert sent to the wrong person could fail to generate help and instead cause negative repercussions if sent to someone who would punish rather than help the adolescent. Although most participants suggested that alerts should be sent to parents or guardians because of their close bonds to their children, parents or guardians also stood out in interviews as both unpredictable and occasionally hurtful. Lucy (age 14) recalled the example of a friend who illustrated this as follows:

...my friend, she’s felt sad before and she even mentioned depression once, and her parents got really mad at her, and they blamed it on her that she was feeling sad and that she mentioned it. So I think that it would be best to tell a teacher versus a parent, or tell emergency services...

John (age 14) similarly noted that “if it were an abusive parent or something like that it wouldn’t be good.” A small number of participants also expressed fear of how their own parents would respond to the alerts.

One common concern was that parents or guardians would not take mental health concerns seriously and may not know how to respond. Lilo (age 17) preferred emergency services over parents since:

self-harm is something that somebody should get treated for and their parent might...be passive about it. Like you never know how serious a parent is going to take it.

Bri (age 17) felt that Latinx families specifically may have limited efficacy in handling mental health concerns:

I feel like it’s different, because of the fact that I know, for Latinos, when there’s mental health included, it’s really hard to talk about it with your parents, especially if your parent is not educated in that, or if your parents is—they’ve grown up in certain ways, and so they will believe that what we think is kind of dumb. Because I know, for some families, they can’t even talk about that type of thing, because it’s seen as a weakness.

Emergency services were more uniformly seen as safe and knowledgeable, but participants felt that not all posts indicating NSSI merited intervention from emergency services. Some participants mentioned a hotline number as an alternative. No participant worried about emergency services introducing risks to their families, although one stated “you wouldn’t want the FBI knocking on your door” [John, age 14].

School staff and teachers were occasionally mentioned as preferable substitutions when participants thought parents or guardians would be unsupportive, but these adults were not immune from concerns about unhelpful responses and punishment. Although privacy concerns for automated alerts in general were minimal, privacy concerns specific to alerts sent to schools were more common. Specifically, some participants worried about other students finding out or that school staff and teachers would judge adolescents for their mental health struggles. Jaden (age 16) explained as follows:

I wouldn’t want that business out there to my school if, you know, right away. Because if they don’t understand the context of the situation, that could just also do the whole labeling thing where it’s like you feel like everyone’s kind of just watching out for you...I wouldn’t want that business there with my school because it’s just, it’s a big place and you’re there for a long time, so it’s kind of like it could have lasting effects on you while you’re there.

Although preferred alert recipients differed across individual adolescents and across perceived severity of situations, participants shared a desire that alerts should be sent to “someone who cares and who wants them to get better” [Sara, age 14]. Overall, the alert recipient was viewed as critical to the efficacy and desirability of alerts derived from social media posts.

## Discussion

### Principal Findings

This study examined the perceptions of Latinx adolescents toward automated alerts for NSSI based on digital phenotyping using social media platforms. Diverging from adult perceptions [27], participants were largely positive toward automated alerts, suggesting that alerts could help overcome the lack of responsiveness from peers and parents. In particular, participants felt that alerts could connect adolescents to help before situations escalated. Notably, the perception that social media users often ignore posts with concerning content rather than report it is among the reasons that Facebook launched its digital phenotyping efforts [13]. Adolescents stressed that alerts must be accurate and directed toward someone able and willing to help. The efficacy of alerts as a mental health intervention is partially contingent on the efficacy of the alert recipient, suggesting the need to adopt practices that accommodate and support the social fabric surrounding adolescents.

The findings reveal opportunities to improve the ethics and efficacy of mental health interventions based on digital phenotyping. With respect to the machine learning algorithms that underpin alerts, the findings show that these must be sensitive to the specific linguistic patterns of adolescents to account for the use of sarcasm, jokes, and hyperbole used for dramatic effect. Furthermore, algorithms must be sensitive to variation across adolescents, including those rooted in cultural differences in the expression of depression and NSSI [35]. Although participants did not suggest that they had concerns unique to their status as Latinx youth, attention should be paid to the specific subpopulations included in the data to train algorithms [36]. Although continued manual screening of posts identified through machine learning, similar to the efforts of Facebook, may help reduce the number of alerts being sent inappropriately, poorly trained machine learning tools are still likely to miss concerning posts. In line with prior research showing that depression can co-occur with NSSI [32,33], interviewees relayed how detecting depression early could offer timely help to limit adolescent harm.

With regard to the actual alerts, the selection of an appropriate recipient is crucial to the adolescent receiving assistance. NSSI represents a particularly interesting scenario for alerts because, unlike suicidality, emergency services are not always the obvious or appropriate choice. Although many participants either conflated NSSI with suicidality or worried about NSSI leading to something more serious, which accords with prior research on Latinx adolescents [31], they felt that emergency services may be an excessive response for NSSI. However, not all adolescents reported access to supportive school staff, teachers, parents, or guardians, and some worried adults would respond in detrimental ways. These concerns are consistent with those found in prior research on adolescents who engage in NSSI and highlight the importance of increased education for adults who surround adolescents [49].

As there was no uniformly appropriate alert recipient across all participants and levels of need, researchers and social media platform developers should consider the development of alerts that take into account the privacy preferences of *individual* adolescents. The lack of uniformly appropriate alert recipients is consistent with a contextual view of privacy [50] and suggests that interventions should be co-designed with adolescents providing feedback to ensure that efforts meet their preferences and needs [51]. In the implementation stage, adolescents should be asked whether they want alerts to be sent for NSSI and, if so, to whom. Engagement is ideally an ongoing process, as privacy preferences and trusted adults may change over time. Allowing participants to opt in to alerts would address one of the major ethical concerns with the current approach of Facebook to digital phenotyping [25].

At the time of writing, we were not aware of any platforms that allow social media users to choose in advance who should receive alerts sent by platforms. Facebook makes use of a two-tiered system in which their machine learning tool identifies users who may be at risk of imminent harm. In the event that Facebook’s Community Operations team agrees with the assessment, the user is shown *support options* [52], including a prompt to reach out to a Facebook friend using prepopulated text. However, the user must decide in that moment to send the message themselves. In the event that the risk to the user is deemed high, the Facebook team instead notifies local emergency services. It should be noted, however, that this system was designed for suicidality rather than NSSI. Furthermore, other platforms popular with adolescents do not have an official alert policy at all, even for suicidality. TikTok, for example, simply states that those considering harming themselves should “please contact [their] local emergency services, a suicide-prevention helpline, or expert listening service for help” [53]. Platforms have room for significant growth in the area of co-designing responses for both NSSI and suicidality. It will also be critical to engage parents, teachers, and emergency service managers in this co-design process to examine their preferences for alerts. In addition, further research is needed to examine and support the capacity of these actors to manage alerts in a constructive manner, especially with respect to recognizing when and how to seek mental health treatment. As noted by the participants themselves, the notification of an adult is not a guarantee that an adolescent will receive mental health services.

Although participants mentioned concerns about their privacy within school settings, none voiced concerns related to social media platforms analyzing or storing mental health relevant information, or ethical concerns about this occurring without their awareness or explicit consent. Furthermore, none expressed concerns about the implications of these data for future employment or insurability [24]. Although there is little research on adolescent perceptions of commercial and institutional privacy [28], these findings are consistent with initial evidence that adolescents are less likely than adults to view their health information as sensitive [29]. It is also possible that the absence of concerns reflects a lack of awareness of risks. Adolescents in the United States may be at particular risk for harms stemming from the intentional or inadvertent disclosure of private health-related information by platforms in light of the power of the private health insurance industry. Although the Affordable Care Act of 2010 currently prevents insurers from denying coverage or charging higher rates due to preexisting conditions, these provisions are currently in jeopardy because of legal challenges [54]. Future research should provide participants with risk information and assess whether support changes for social media platforms engaging in digital phenotyping. Any effort by social media companies to expand current efforts should adopt a digital literacy component to achieve meaningful informed consent. More broadly, it is notable that Facebook’s automated suicide-prevention feature is not available in the European Union because the General Data Protection Regulation provides social media users greater control over their *data concerning health* [12,55]. The United States currently has no equivalent law, and the Health Insurance Portability and Accountability Act applies only to health care providers, plans, and clearinghouses [55]. Further work is needed to ensure that social media platforms protect the health-related information they gather and analyze, particularly concerning minors.

Overall, the findings suggest that automated alerts based on digital phenotyping of social media data may offer opportunities to address NSSI among Latinx adolescents and prevent further harm. This is a significant finding given the high rates of NSSI and barriers to receiving mental health treatment among Latinx adolescents [2,35,56]. Further, Latinx adolescents have a high rate of smartphone access and report high levels of social media use [57], indicating a wide range of opportunities for deployment. However, the implementation of alerts without addressing underlying disparities in access to mental health services in the United States may exacerbate disparities because such interventions risk being more effective for populations with access to care [37,58-60]. In addition to practical concerns with regard to algorithms and alert recipients, developers should also be mindful of the broader socioeconomic implications of their efforts.

### Limitations

With regard to limitations, the transferability of findings should be considered in light of the fact that this study was conducted with English-speaking Latinx adolescents drawn from a summer program in a midsized US city that is largely of Mexican origin [43]. The homogeneous sample helps address the underrepresentation of Latinx groups in privacy research and their disproportionate risk of NSSI. Further research is needed to reveal how concerns and preferences may be distinct across populations. In addition, as this study was one element within a larger project, time constraints prevented additional questions prompting participants to reflect explicitly on questions of commercial privacy. However, the absence of probing allowed us to capture the degree to which participants readily reflected on these concerns. It should also be stressed that participants were not selected based on prior experiences with NSSI, which allows findings to be transferable to a broader pool of adolescents but may fail to capture the unique preferences of those engaging in NSSI. In light of prior research indicating that many adolescents choose to seek help for NSSI on the internet [61], and that these adolescents may experience more suicidality than those who do not seek help on the internet [62], there is a strong need for additional work exploring whether the benefits of NSSI alerts would offset any potential chilling effects on help seeking via social media. Finally, future research should also expand beyond adolescents to consider the capacity and preferences of adults and institutions who could serve as alert recipients.

### Conclusions

This research finds broad support among Latinx adolescents for automated alerts issued in response to NSSI posts on social media, indicating an opportunity for deploying interventions grounded in digital phenotyping to address mental health on the internet. Latinx adolescents described pragmatic concerns about the accuracy of these tools and who should receive alerts but had few ethical concerns and expressed little awareness of the risks posed by the collection and storage of health-related information by commercial entities. Researchers and social media platforms should collaborate with adolescents to co-design interventions that accurately and appropriately alert a chosen recipient when they are at risk for NSSI. Any such effort should consider the implications for adolescent privacy. Ensuring access to appropriate mental health services following detection will be critical to avoid exacerbating disparities.

## Abbreviations

NSSI: nonsuicidal self-injury
